# Oridonin Attenuates Synaptic Loss and Cognitive Deficits in an Aβ_1–42_-Induced Mouse Model of Alzheimer’s Disease

**DOI:** 10.1371/journal.pone.0151397

**Published:** 2016-03-14

**Authors:** Sulei Wang, Linjie Yu, Hui Yang, Chaosheng Li, Zhen Hui, Yun Xu, Xiaolei Zhu

**Affiliations:** 1 Department of Neurology, Nanjing Drum Tower Hospital Clinical College of Traditional Chinese and Western Medicine, Nanjing University of Chinese Medicine, Nanjing, PR China; 2 Department of Neurology, Affiliated Drum Tower Hospital of Nanjing University Medical School, Nanjing, PR China; 3 Jiangsu Stroke Research Collaborative Group, Nanjing, PR China; 4 Jiangsu Province Stroke Center for Diagnosis and Therapy, Nanjing, PR China; 5 Jiangsu Key Laboratory for Molecular Medicine, Nanjing University Medical School, Nanjing, PR China; Okayama University Graduate School of Medicine, Dentistry and Pharmaceutical Sciences, JAPAN

## Abstract

Synaptic loss induced by beta-amyloid (Aβ) plays a critical role in the pathophysiology of Alzheimer’s disease (AD), but the mechanisms underlying this process remain unknown. In this study, we found that oridonin (Ori) rescued synaptic loss induced by Aβ_1–42_ *in vivo* and *in vitro* and attenuated the alterations in dendritic structure and spine density observed in the hippocampus of AD mice. In addition, Ori increased the expression of PSD-95 and synaptophysin and promoted mitochondrial activity in the synaptosomes of AD mice. Ori also activated the BDNF/TrkB/CREB signaling pathway in the hippocampus of AD mice. Furthermore, in the Morris water maze test, Ori reduced latency and searching distance and increased the number of platform crosses in AD mice. These data suggest that Ori might prevent synaptic loss and improve behavioral symptoms in Aβ_1–42_-induced AD mice.

## Introduction

Alzheimer’s disease (AD) is the most common cause of dementia in the elderly and is a prevalent neurodegenerative disease characterized by neuronal loss, extracellular senile plaques and intracellular neurofibrillary tangles. In 2015, an estimated 5.3 million people have AD in United States, and AD makes the sixth leading cause of death[1]. Although the underlying mechanisms of AD have not been fully elucidated, converging lines of evidence indicate that the accumulation of beta-amyloid (Aβ) is the key event in AD pathology. Aβ peptides are produced from the consecutive cleavage of β-amyloid precursor protein (APP) by β-secretase and γ-secretase, and Aβ_1–42_ is considered as the major neurotoxic Aβ species in the brain[2,3]. Genetic evidence has established the causative role of Aβ generation in AD pathogenesis, and this amyloidogenic processing of APP might be linked to essential cellular processes uniquely disrupted in AD brains[4,5]. The deposition of Aβ affects the morphology and function of synapses by disrupting the synaptic signaling pathways and destroys dendritic spines, which leads to deficits in memory and behavior[6]. In addition, brain tissues from AD patients show a marked loss of synapses, which could underlie the observed cognitive decline[7]. Emerging evidence also suggests that protecting synapses could attenuate the observed memory deficits in animal models of AD[8,9], which indicates that new approaches that target synapses could provide disease-modifying therapeutics.

Synaptic dysfunction in AD has been extensively studied, and the results suggest a central role for the synapse in AD pathogenesis. However, the exact mechanisms by which Aβ specifically targets synapses remain largely unknown. Brain-derived neurotrophic factor (BDNF), a member of neurotrophin family, plays a critical role in synaptic regulation and is thought to ensure synaptic growth, promote synaptic transmission and enhance synaptic plasticity[10]. A reduction in BDNF expression has been found in the hippocampus of AD mice[11]. In addition, Aβ significantly decreases the levels of BDNF in dendritic cells derived from AD patients[12]. The biological function of BDNF is regulated by its high-affinity receptor, tropomyosin-related kinase B (TrkB), which is known to be coupled to the activation of the phosphatidylinositol 3-kinase/Akt, phospholipase C-γ (PLC-γ) and Ras/ERK pathways[13]. Aβ decreases TrkB expression in the hippocampal dentate gyrus of Aβ-injected rats[14], and activating TrkB protects neurons against Aβ toxicity[15]. cAMP response element-binding protein (CREB), a downstream target of this pathway, is strongly related to the maintenance and activity of synapses and the formation of memories[16–18]. Taken together, these results support the hypothesis that the BDNF/TrkB/CREB pathway is involved in the pathological processes of AD.

Oridonin (Ori), isolated from the traditional Chinese herb Rabdosia rubescens, is an active diterpenoid which is a trepenoid with the C_20_ skeleton, Ori has diverse pharmaceutical and biological factions and has already been used in clinical practice. We have previously reported that Ori suppresses Aβ-induced neuroinflammation by inhibiting the NF-κB pathway[19]. Thus, in this study, we show the effects of Ori on Aβ_1–42_-induced synaptic loss and investigate the molecular mechanisms involved in the protection of synapses.

## Materials and Methods

### Reagents and antibodies

Human Aβ_1–42_ was purchased from EMD Millipore Corporation (Billerica, MA, USA). Ori was obtained from Chengdu Must Bio-Technology Company (Sichuan, China). Neurobasal media and B27 were bought from Life Technologies Corporation. MAP-2 antibody was purchased from Abcam (Cambridge, MA, USA). COX-2 antibody was bought from Santa Cruz Biotechnology (Santa Cruz, USA). PSD-95 antibody, synaptophysin antibody and CREB antibody were obtained from CST (Cell Signaling Technology, USA). 4', 6'-diamidino-2-phenylindole (DAPI), VDAC1, BDNF, p-TrkB, TrkB, p-CREB and β-actin antibodies were purchased from Bioworld Technology (Bioworld, USA). Lamin B1 antibody and HRP-conjugated secondary antibodies were also obtained from Bioworld.

### Animals

Experiments were conducted using male C57BL/6 (B6) mice weighing 20–26 g. This study was reviewed and approved by the Animal Care Committee of Nanjing University. We made every effort to minimize the number of mice used and their suffering, and no mice died through the experiment. Mice were housed in specific pathogen free (SPF) cages under standard laboratory conditions including 21±1°C temperature, 40–55% relative humidity and 12-h light/dark cycle with free access to water and food. All mice in the study were sacrificed after the treatment. The mice were anesthetized with sodium pentobarbital (50 mg/kg) and decapitated, and brain tissues were removed for the western blot and synaptosomes study. In the immunostaining study, the mice were anesthetized and perfused intracardially with 0.9% saline followed by 4% paraformaldehyde. The brains were removed and sequentially post-fixed in 15% and 25% sucrose solutions. The experiments were performed in a double-blinded manner.

### AD mouse model and treatment with Ori

Aβ_1–42_ was prepared with 1% NH_3_xH_2_O at a concentration of 1 mg/ml and incubated at 37°C for 5 days to allow for oligomerization as described previously[20]. Ori was dissolved in DMSO at a concentration of 20 mg/ml and diluted to the desired concentration in saline as described previously[19]. The i.c.v microinjections of Aβ_1–42_ (4 μg) into the bilateral hippocampus of male C57BL/6 (B6) mice were given via infusion cannulae as previously described[21]. After injecting Aβ_1–42_ for 7 days, Ori was injected intraperitoneally once a day for 15 days. Ori (10 mg/kg/day, i.p. for 15 days) attenuated memory deficits in Aβ_1–42_ induced AD mice, but it did not exert the protective effects at higher doses (20–50 mg/kg/day)[19]. Thus, the dose of 10 mg/kg/day intraperitoneally injected for 15 days was used in this study. Mice were divided into the following groups: control mice (sham-operated) that received saline; control mice (sham-operated) that received Ori (10 mg/kg/day, i.p. for 15 days); Aβ_1–42_-induced AD mice that received saline; and Aβ_1–42_-induced AD mice that received Ori (10 mg/kg/day, i.p. for 15 days). At the end of the treatment, mice were behaviorally tested and sacrificed for the following experiments.

### Cell culture and stimulation

Primary cortical neurons were derived from the brains of E15-17 C57BL/6 (B6) mouse embryos as previously described[22]. Briefly, the cerebral cortex was isolated, trypsinized in 0.25% trypsin at 37°C for 10 min and seeded onto 24-well poly-D-lysine-coated plates with cover slips at a density of 5 × 10^5^ cells/ml. Cells were cultured in neurobasal media supplemented with B27 and 25 nmol/L glutamine at 37°C in a humidified 5% CO_2_ incubator. Neurons were maintained in the media for 8 days, and the purity of the primary neurons was more than 95%. Neurons were pre-treated with Ori (10–100 μM) for 1 h and 2 μM Aβ_1–42_ for 6 h, and then prepared for the following experiments. Our preliminary data indicated that Ori (40 μM) significantly increased the viability of neurons treated with Aβ_1–42_, while the doses of more than 40 μM did not exert the protective effects (data not shown), and the dose of 40 μM was used in this study.

### Western blotting

Three animals from each group were euthanized and decapitated, and the hippocampus of the mice were removed and sonicated in RIPA buffer (Beyotime Institute of Biotechnology, China) containing phosphatase and protease inhibitor cocktails (Sigma-Aldrich, USA). The total protein was obtained from the supernatant of this extract following 30 min of centrifugation at 12000×rpm, and the protein content was quantified by the BCA protein assay kit (Bioworld, USA). Western blotting was performed as described previously[23]. Samples (30 μg protein) were separated by SDS-PAGE and electrophoretically transferred onto PVDF membranes. Membranes were blocked for 1 h in 5% non-fat milk and incubated overnight with primary antibodies. β-actin was used as a loading control. The incubation with primary antibodies was followed by a 2 h incubation with HRP-conjugated anti-rabbit or anti-mouse secondary antibodies. The protein signals were detected using the chemiluminescence reagents provided with the ECL kit (Bioworld, USA), and Image J software was used to determine the intensity of the blots.

### Immunostaining

Immunostaining was performed as described previously[24]. After being anesthetized, mice were perfused intracardially with 0.9% saline followed by 4% paraformaldehyde. The brains were removed and sequentially post-fixed in 15% and 25% sucrose solutions. Subsequently, the brains were cut into 15 μm sections using a cryostat microtome (Leica, Germany). After washing in phosphate buffered saline (PBS) three times for 10 min each and blocking in 3% BSA for 1 h at room temperature, the sections were incubated overnight with an anti-BDNF antibody at 4°C. After washing in PBS three times for 10 min each, the samples were incubated with the secondary antibody for 1 h at 37°C in the dark. Subsequently, the cell nuclei were labeled by DAPI. Images were detected using an OLYMPUS BX51 microscope (Olympus, Japan).

For double immunostaining, cells were washed three times with PBS and then fixed in 4% paraformaldehyde for 30 min at room temperature. Subsequently, cells were blocked with 3% BSA for 60 min. After an overnight incubation with anti-PSD-95 antibody or anti-synaptophysin antibody and MAP-2 antibody at 4°C, the cover slips were washed for 10 min three times and then incubated with goat anti-rabbit and goat anti-mouse antibodies for 1 h at 37°C in the dark. DAPI was used to stain the cell nuclei, and images were acquired using an OLYMPUS BX51 microscope. The immunostaining analysis was performed as described previously[25]. All procedures were conducted in a randomized and blinded manner.

### Morris water maze (MWM) test

Cognitive function was evaluated by the Morris water maze as previously described[23]. Briefly, the test involved 5 days of acquisition training with four trials per day. For each trial, each mouse was given 60 s to find a platform that was submerged 2 cm underwater in a circular black pool; if the mouse failed to find the platform within this period, they were manually placed on the platform for 10 s, and the latency was recorded for 60 s. The escape distance, escape latency and swimming speed were analyzed by Any-maze software (Stoelting, USA). On the 6th day, the hidden platform was removed from the water maze, and mice were allowed to swim freely for 60 s; the number of times the mouse crossed the target platform were recorded. The observers were blind to the experimental conditions.

### Synaptosomal preparation

To obtain synaptosomes, mice were anesthetized, and their brains were removed, weighed and dounced in a glass Teflon homogenizer in 10 vol (1:10, wt/vol) of Syn-PER synaptic protein extraction reagent (Thermo, Rockford, USA) supplemented with a protease and phosphatase inhibitor cocktail. Following the manufacturer’s instructions, the homogenate was centrifuged at 1200g for 10 min at 4°C, and then the supernatant was centrifuged for a further 20 min at 15000g at 4°C. The supernatant (cytosolic fraction) was removed, and the synaptosome pellets were resuspended in Syn-PER reagent. The protein concentration of synaptosomes was determined by the BCA method.

### Synaptosome activity assay

The synaptosome activity assay was performed as described previously[26]. The relative amount of mitochondria was determined by western blot with VDAC1 as a loading control. The mitochondrial activity, an index of the functional status of the synaptosomes, was assessed by the conventional 3-[4, 5-diethylthiazol-2-yl]-2,5-diphenyltetrazolium bromide (MTT) assay. Briefly, 2.0 mg of synaptosome protein was prepared, and MTT solution (5 mg/ml in PBS) was added and incubated at 37°C for 3 h. Subsequently, formazan crystals were dissolved in DMSO, and the absorbance was read at 570 nm by a microplate reader.

### Golgi staining

The morphology of dendritic spines in the brain was analyzed using a FD Rapid GolgiStain Kit (FD Neurotechnologies, Elliot City, MD, USA) according to the manufacturer’s instructions. Briefly, after being anesthetized, the mouse brains were quickly removed and washed in double distilled water (DDW). Subsequently, the brains were submerged in impregnation solution for 2 weeks at room temperature in the dark. The impregnation solution was replaced on the next day. Samples were removed to solution C for 2 days at room temperature in the dark, sectioned into 200 μm sections by a cryostat microtome and placed onto microscope slides. Sections were washed three times for 10 min each and placed in a mixture composed of solution D, solution E and DDW for 10 min followed by two rinses in DDW for 4 min each. The slides were dehydrated, cleared and coverslipped.

### Analysis of dendritic morphology

Dendritic morphology was measured by an investigator blind to the experimental conditions using an OLYMPUS BX51 microscope (Olympus, Japan). Golgi-stained brains from 6 mice from each group were subjected to morphological analysis, and 6 neurons from the hippocampus of each brain were selected randomly for analysis. In addition, the spine density was observed under a 100x oil immersion and was expressed as the number of spines per 10 μm of dendrite. The total dendritic length, numbers of branches and spine density were measured by Image J software.

### Statistical analysis

All data are presented as the means ± SEM and analyzed using SPSS version 13.0 (SPSS, Chicago, IL, USA). For the MWM tasks, group differences in the escape latency, searching distance and swimming speed during the MWM test were analyzed using two-way analysis of variance (ANOVA) with repeated measures followed by Bonferroni multiple comparison test with day and treatment as the sources of variation. All other data were analyzed with a one-way ANOVA followed by Bonferroni’s post hoc. P-values smaller than 0.05 (P<0.05) were considered statistically significant.

## Results

### Ori increases the expression of PSD-95 and synaptophysin in the hippocampus of AD mice

**Fig 1:**
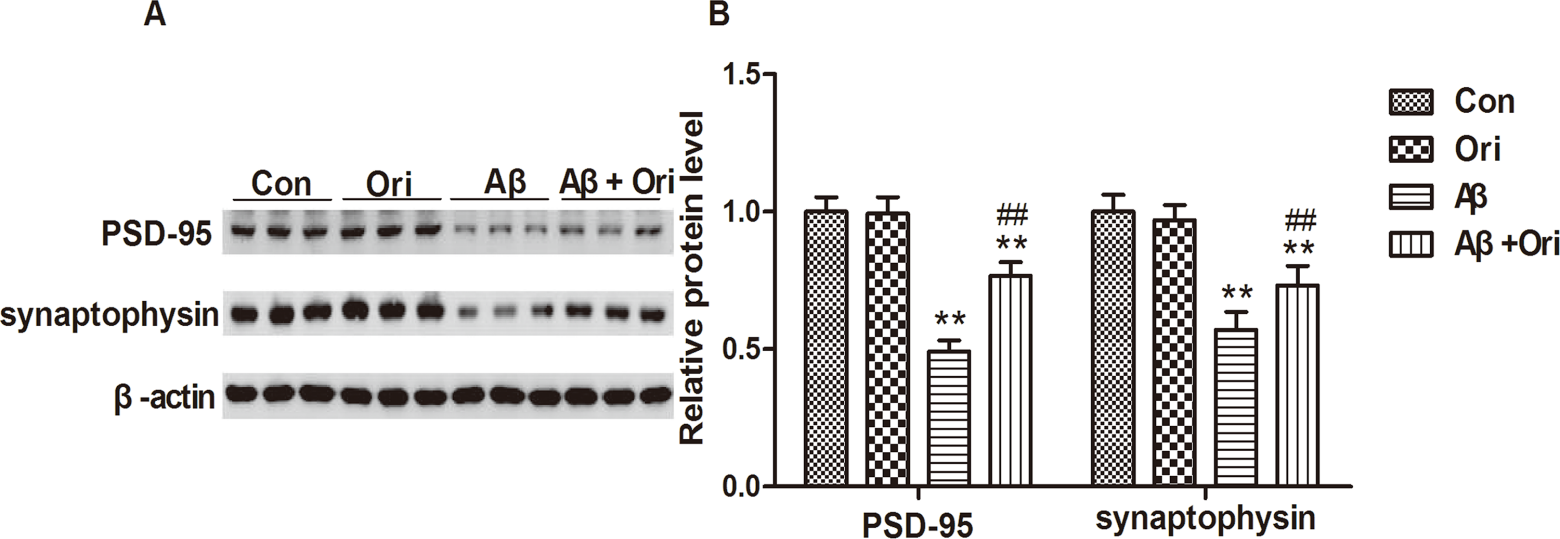
Ori increases the expression of PSD-95 and synaptophysin in the hippocampus of AD mice. (A) Western blotting for PSD-95 and synaptophysin proteins. (B) Quantitative analysis of PSD-95 and synaptophysin expression. The relative expression levels of PSD-95 and synaptophysin were normalized against β-actin and are presented as the ratio of the values of the experimental group to the control group. n = 5 mice per group and the experiment was performed for three times. Data are presented as the means ± SEM. *P<0.05, **P<0.01 vs. control; #P<0.05, ##P<0.01 vs. Aβ_1–42_.

Synaptic loss is one of the neuropathological hallmarks of AD, and synaptophysin and PSD-95 are the markers of the pre- and post-synapse, respectively. Compelling evidence has shown that the levels of PSD-95 and synaptophysin are reduced in AD transgenic mouse models[27,28] and in brains from AD patients[29]. In this study, we first tested the expression of PSD-95 and synaptophysin in the hippocampus of AD mice. As shown in Fig 1, the western blot analysis revealed significant reductions of PSD-95 and synaptophysin expression in the hippocampal samples. However, after Ori treatment, the expressions of PSD-95 and synaptophysin were significantly increased compared to the AD group. These data indicated that Ori attenuated the loss of synaptic proteins that was induced by Aβ *in vivo*.

### Ori promotes the expression of PSD-95 and synaptophysin in Aβ_1–42_ treated neurons

**Fig 2:**
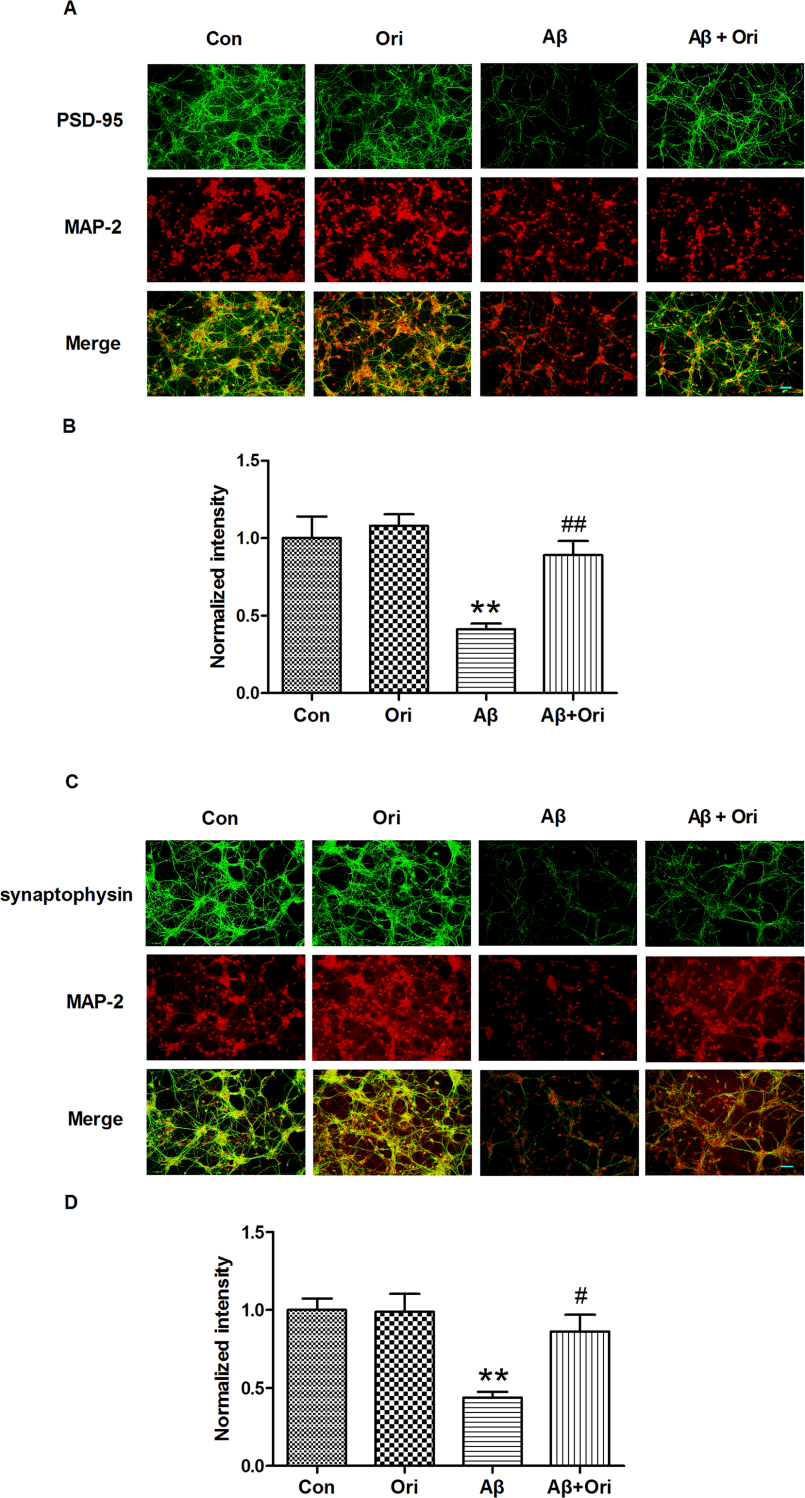
Ori promotes the expression of PSD-95 and synaptophysin in Aβ_1–42_-treated neurons. (A) Immunostaining for PSD-95 in Aβ_1–42_-treated neurons. (B) Quantitative analysis of PSD-95 staining. (C) Immunostaining for synaptophysin in Aβ_1–42_-treated neurons. (D) Quantitative analysis of synaptophysin staining. Data are presented as the means ± SEM and normalized to control neurons. *P<0.05, **P<0.01 vs. control; #P<0.05, ##P<0.01 vs. Aβ_1–42_. Scale bar = 50 μm. All measurements were made by blinded investigators, and the final quantification was based upon at least three independent experiments.

To explore whether Ori could rescue PSD-95 and synaptophysin expression in Aβ_1–42_-treated neurons, we observed the expression of PSD-95 and synaptophysin by immunostaining. As shown in Fig 2, the levels of PSD-95 (Fig 2A and 2B) and synaptophysin (Fig 2C and 2D) were significantly decreased in the AD group, and this reduction was partially reversed by Ori treatment. Thus, our results indicated that Ori attenuated synaptic loss *in vivo* and *in vitro*.

### Ori rescues the dendritic morphological changes in the hippocampus of AD mice

**Fig 3:**
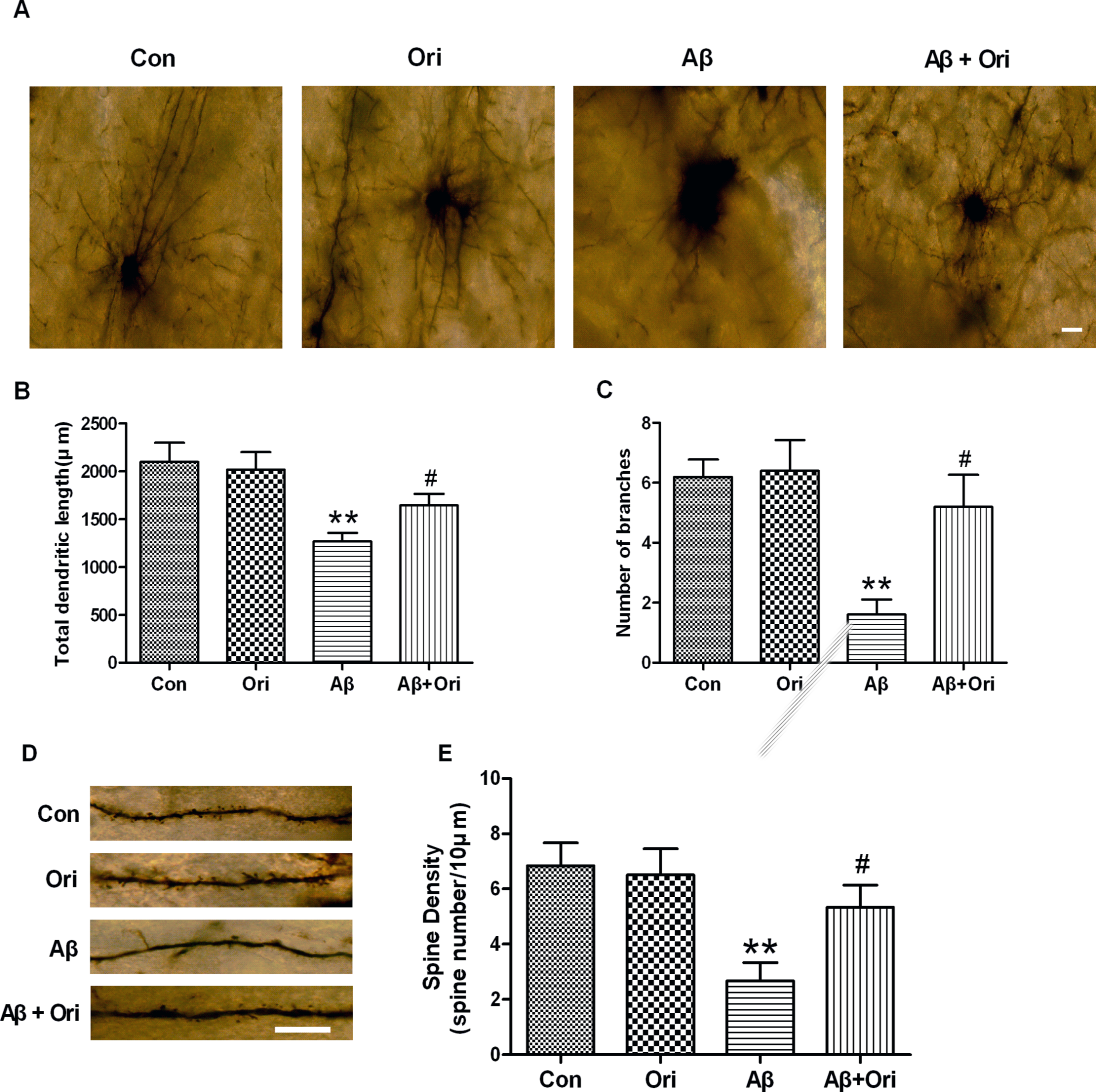
Effects of Ori on the total dendritic length, number of branches and spine density in the hippocampus of AD mice. (A) Representative dendritic images from the hippocampus of mice. Scale bar = 20 μm. (B) Quantitative analysis of the total dendritic length. (C) Quantitative analysis of the number of branches. (D) Representative dendritic spine images from the hippocampus of mice. Scale bar = 10 μm. (E) Quantitative analysis of spine density. Six neurons from each brain were selected for analysis and 6 mice were used from each group. Data are presented as the means ± SEM. *P<0.05, **P<0.01 vs. control; #P<0.05, ##P<0.01 vs. Aβ_1–42_.

The development of dendrites and the number of dendritic spines, which are compromised in AD mice, can both affect synaptic function[30]. Here, we used Golgi staining to examine the effects of Ori on dendritic morphology. As shown in Fig 3A, the total dendritic length and the number of branches did not differ between control and Ori-injected mice. Aβ significantly decreased the total dendritic length compared to control mice, while Ori-treated AD mice had significantly longer dendrites compared to untreated AD mice. Moreover, AD mice showed a reduction in the number of dendritic branches, whereas the number of branches was markedly increased following Ori treatment. To further characterize the differences in dendritic morphology between groups, we examined the density of dendritic spines. The hippocampus of AD mice exhibited a lower density of spines, and this reduction in spine density could be rescued by Ori (Fig 3D).

### Ori increases the expression of PSD-95 and synaptophysin and promotes mitochondrial activity in the synaptosomes of AD mice

**Fig 4:**
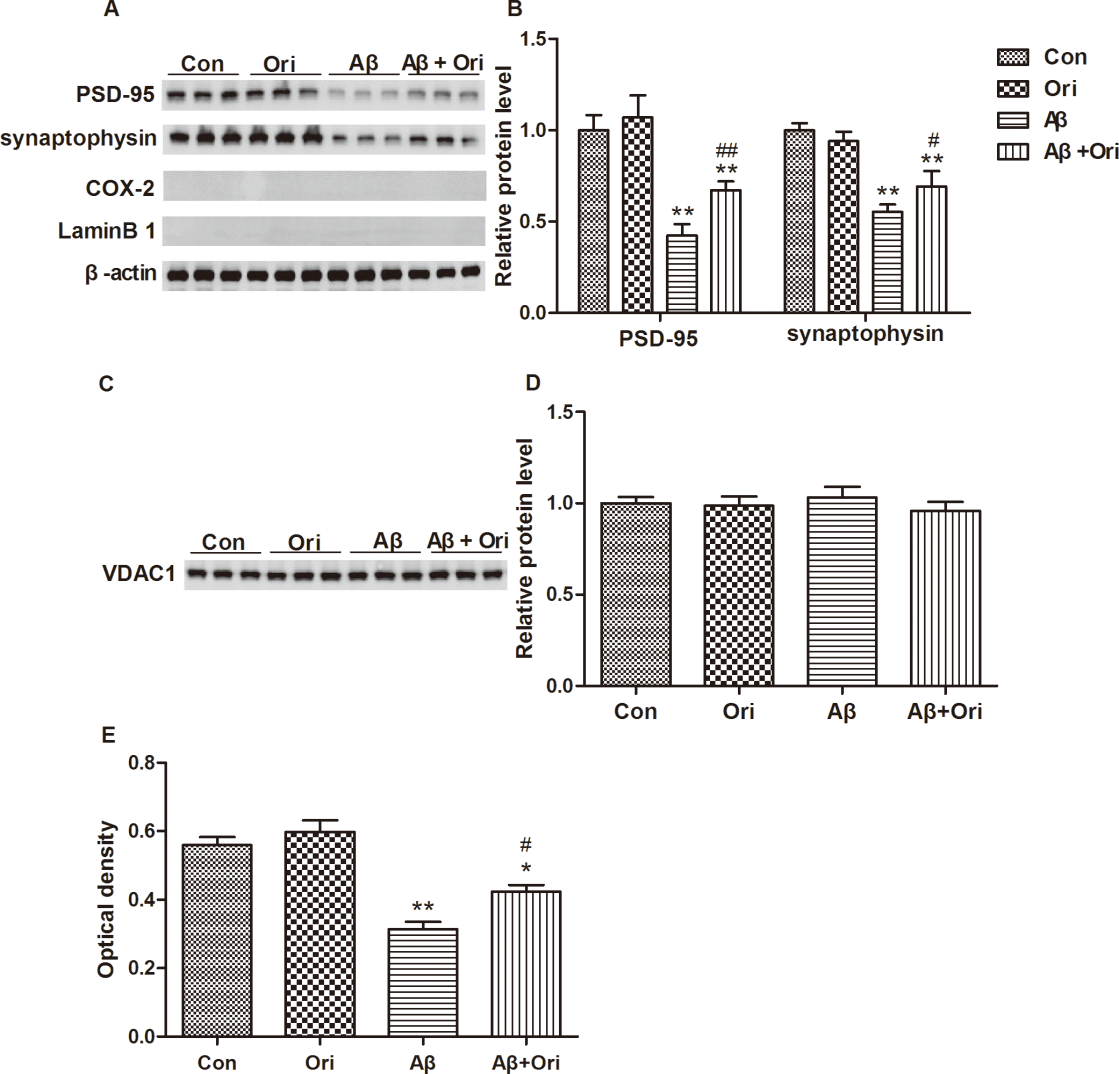
Ori increases the expression of PSD-95 and synaptophysin and promotes mitochondrial activity in the synaptosomes of AD mice. (A) Western blotting for PSD-95, synaptophysin, COX-2 and Lamin B1 proteins. The absence of the cytosolic protein COX-2 and the nuclear protein Lamin B in the synaptosome pellets confirms the purity of synaptosomes. (B) Quantitative analysis of PSD-95 and synaptophysin expression. The relative expression levels of PSD-95 and synaptophysin were normalized against β-actin and are presented as the ratio of the values of the experimental group to the control group. (C) Western blotting for VDAC1 protein. (D) Quantitative analysis of VDAC1 expression. (E) MTT assay for mitochondrial activity of synaptosomes. n = 5 mice per group and the experiment was performed for three times. Data are presented as the means ± SEM. *P<0.05, **P<0.01 vs. control; #P<0.05, ##P<0.01 vs. Aβ_1–42_.

Synaptosomes, which are isolated synaptic terminals from neurons, can provide material for protein experiments and studies of the maintenance of metabolic activity. We also examined the levels of PSD-95 and synaptophysin in synaptosomes. As shown in Fig 4A and 4B, Ori significantly upregulated the expression of PSD-95 and synaptophysin in the synaptosomes, which was consistent with our results shown in Fig 1. Given that there were no significant differences in the levels of VDAC1 (Fig 4C and 4D), we next measured mitochondrial activity via the MTT assay. As shown in Fig 4E, mitochondrial activity was significantly reduced in synaptosomes from AD mice compared to controls. However, treatment with Ori ameliorated the Aβ-induced reduction in mitochondrial MTT. These data also demonstrated that Ori exerted protective effects on the viability of synapses.

### Ori treatment enhances the BDNF/TrkB/CREB signaling pathway in the hippocampus of AD mice

**Fig 5:**
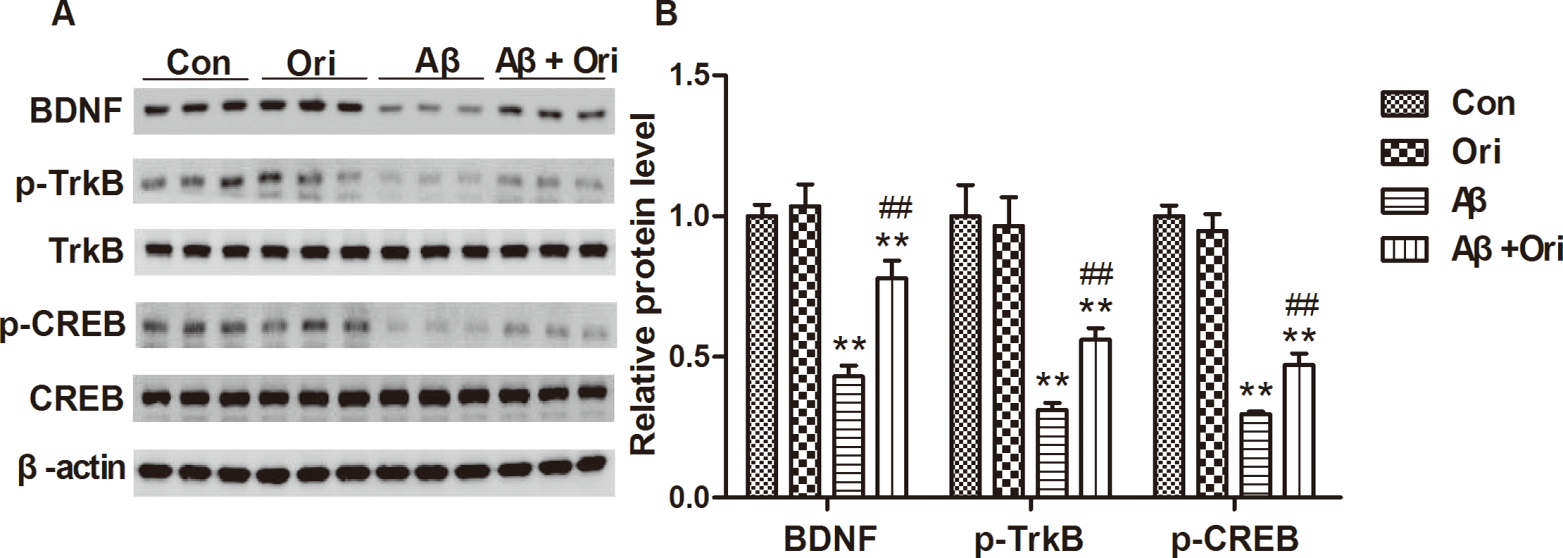
Ori activates the BDNF/TrkB/CREB signaling pathway in the hippocampus of AD mice. (A) Western blotting for BDNF, p-TrkB, TrkB, p-CREB and CREB proteins. (B) Quantitative analysis of BDNF, p-TrkB and p-CREB expression. n = 5 mice per group and the experiment was performed for three times. Data are presented as the means ± SEM. *P<0.05, **P<0.01 vs. control; #P<0.05, ##P<0.01 vs. Aβ_1–42_.

**Fig 6:**
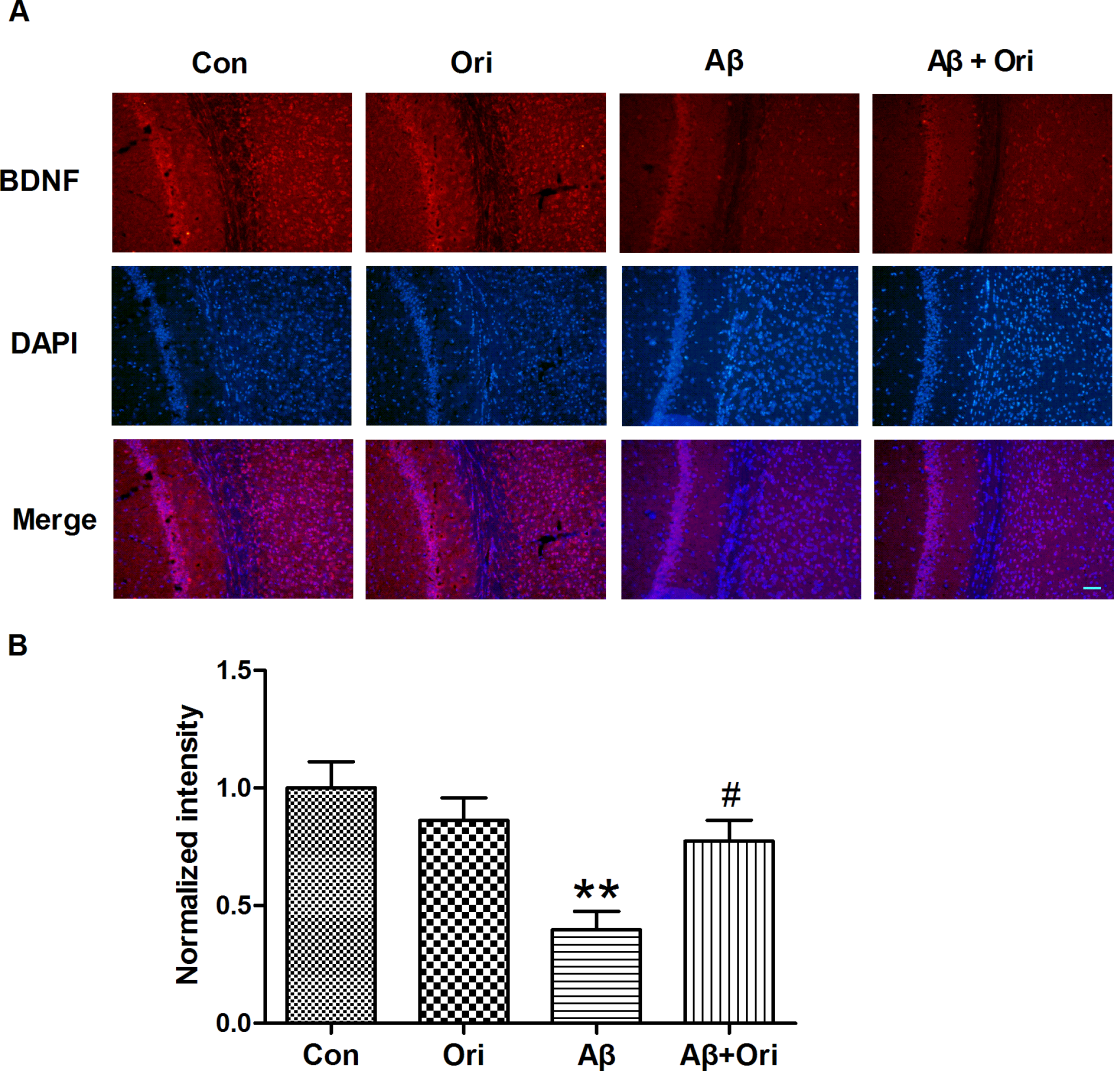
Ori increases the expression of BDNF in the hippocampus of AD mice. (A) Immunostaining for BDNF in the hippocampus. (B) Quantitative analysis of the expression of BDNF normalized to the control group. n = 5 mice per group and the experiment was performed for three times. Data are presented as the means ± SEM. *P<0.05, **P<0.01 vs. control; #P<0.05, ##P<0.01 vs. Aβ_1–42_. Scale bar = 50 μm.

Next, we investigated whether Ori activated the BDNF/TrkB/CREB signaling pathway. As shown in Fig 5, the levels of BDNF, p-TrkB and p-CREB were decreased in the hippocampus of AD mice compared to control mice, and the levels of all three proteins were significantly upregulated after Ori treatment. Our immunostaining also indicated that the reduction in BDNF expression was reversed by Ori (Fig 6). These results showed that the activation of the BDNF/TrkB/CREB signaling pathway might participate in the neuroprotective effects mediated by Ori.

### Ori improves cognitive deficits in Aβ_1–42_-induced AD mice

**Fig 7:**
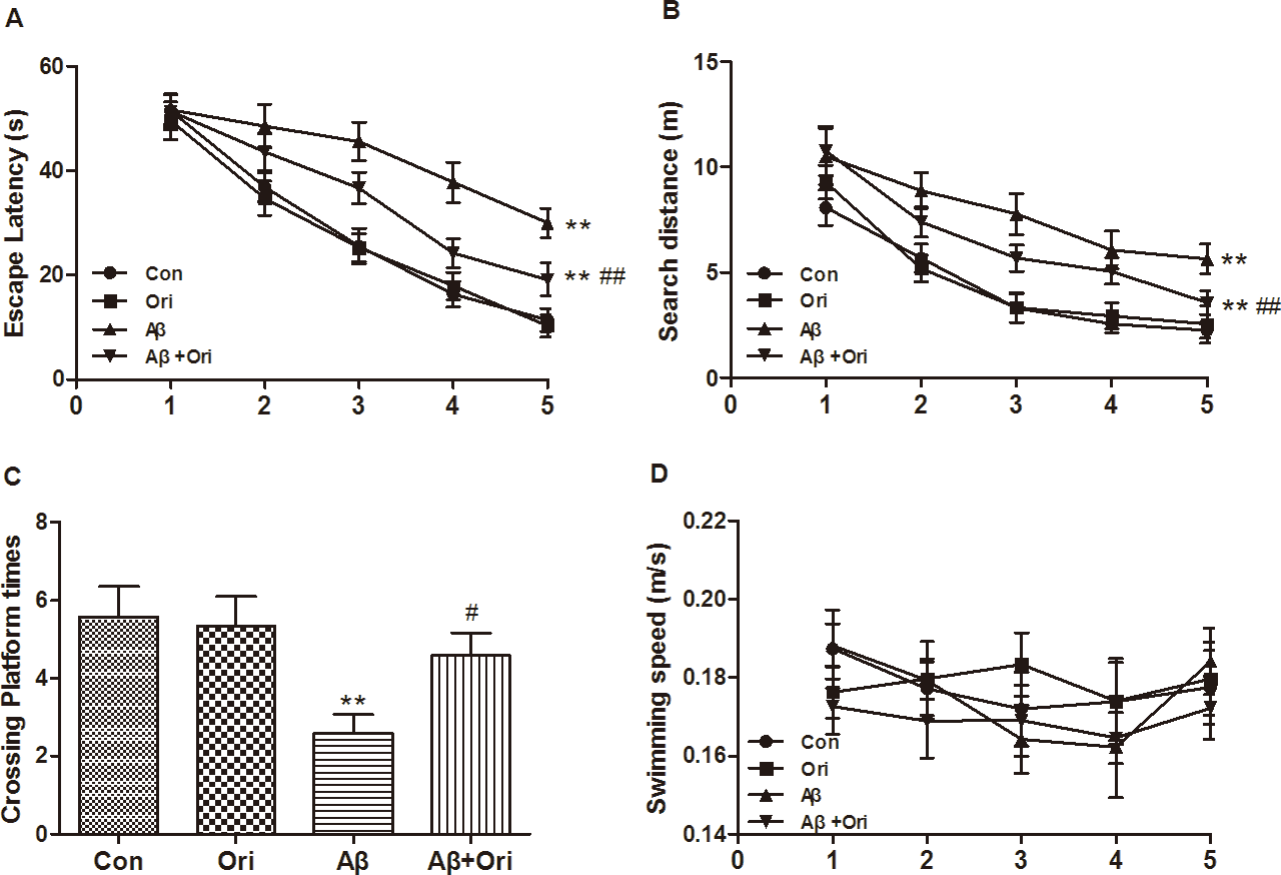
Ori improves cognitive deficits in Aβ_1–42_-induced AD mice. (A) Escape latency, (B) search distance, (C) the number of platform crosses and (D) swimming speed in the Morris water maze. n = 10 mice per group and the experiment was performed for three times. Data are presented as the means ± SEM. *P<0.05, **P<0.01 vs. control; #P<0.05, ##P<0.01 vs. Aβ_1–42_.

Finally, to examine whether Ori improved the cognitive deficits induced by Aβ, we measured the therapeutic potential of Ori to reverse AD-induced declines in spatial memory and learning ability by the Morris water maze. The AD group showed a longer escape latency compared to the control group. However, the reduced latency induced by Aβ was ameliorated by the administration of Ori (Fig 7A). The increase in searching distance induced by Aβ was also improved following Ori treatment (Fig 7B). On the 6th day of testing, the hidden platform was removed from the pool, and all groups of mice were allowed to swim for 1 min. The number of platform crosses by Ori-treated mice was dramatically increased compared to AD mice (Fig 7C). Throughout the entire trial, no significant differences in swimming speed were observed across the groups (Fig 7D), suggesting that there were no differences in motivation. The results from the MWM tests indicated that Ori improved the spatial learning and memory disorders in AD mice.

## Discussion

Aβ plays a critical role in producing the cognitive dysfunction and other characteristics of AD, including synaptic loss, neuroinflammation, neuronal apoptosis, and neurofibrillary tangles. However, the underlying mechanism for Aβ-mediated synaptic loss is not yet fully understood. In current study, we demonstrated that Ori activated the BDNF/TrkB/CREB signaling pathway, prevented synaptic loss and dysfunction and ameliorated cognitive deficits in Aβ-induced AD mice.

Although the exact cause of AD is poorly understood, the production and deposition of Aβ is widely believed to be involved in its pathogenesis. The essential role of Aβ suggests that therapies that target Aβ might be promising for the treatment of AD. Because the major form of amyloid deposition is primarily Aβ_1–42_[31] and because the levels of Aβ_1–42_ are thought to be a potential differentiator between AD and non-AD dementias[32], we used the oligomerization of Aβ_1–42_ *in vivo* and *in vitro*. In AD, Aβ-induced synaptic dysfunction and loss is an important event that is associated with memory deficits, and a reduction of synapses and decrease in synaptic markers have been widely reported in early and late stages of AD[33–37]. Aβ exerts its synaptotoxicity by binding to synapses, which leads to suppression of long-term potentiation (LTP), impairments in synaptic plasticity and neuronal dysfunction[38]. In addition, the Aβ-induced loss of synapse number occurs in a dose-dependent manner[39]. In this study, PSD-95 and synaptophysin were significantly reduced in Aβ-induced AD mice; however, this loss was attenuated by Ori treatment, which suggested that Ori prevented Aβ-induced synaptic loss. Additionally, Ori increased the expression of PSD95 and synaptophysin in Aβ-treated primary cortical neurons. In agreement with the hypothesis that abnormal dendritic morphology is involved in the process of synaptic dysfunction in transgenic mouse models of AD[40–42], we found that Aβ negatively impacted dendritic morphology in AD mice. Meanwhile, Ori increased the overall dendritic length and number of branches and enhanced the number of dendritic spines, which favored the improvement of synaptic function and cognitive deficits.

In recent years, synaptosomes, which are isolated from synapses, have been widely used for the study of AD synapses[43,44]. Indeed, both structural and functional studies of the synapse are supported by synaptosome preparation because they can be stimulated to release neurotransmitters and supply material for protein studies and reflect synaptic function[45]. Studies from post-mortem human brains show that Aβ and p-tau are accumulated in synaptosomes[46,47]. Moreover, the levels of oxidative markers are significantly increased in the synaptosomes from AD postmortem frontal cortices, which implicates the participation of oxidative stress in synaptic loss[48]. Nicotinamide and 3-aminobenzamide exert neuroprotective effects in Aβ-induced AD rats by improving mitochondrial function in synaptosomes[49]. Our current study showed that Ori increased the expression of PSD-95 and synaptophysin in the synaptosomes of AD mice. We also analyzed mitochondrial activity, which was an index of synaptosomal functional integrity, and found Ori improved the mitochondrial activity in synaptosomes from the brains of AD mice. This suggested that Ori exerted protective effects of synaptosomes.

BDNF and its receptor TrkB are widely expressed in the brain, and the binding of BDNF to TrkB regulates the survival and differentiation of neurons and modulates long-term potentiation and plasticity[50,51]. In addition, the BDNF/TrkB signaling pathway is essential for modulating learning and memory[52]. Emerging evidence shows that abnormal BDNF/TrkB signaling is associated with the progression of AD and is involved in the production of Aβ, tau hyperphosphorylation and cognitive impairment[53]. BDNF/TrkB signaling pathway has also been implicated in the morphogenesis, strength, activity and plasticity of synapses, which suggests its crucial roles during various phases of synaptic development[54]. This study confirmed that Ori activated the BDNF/TrkB pathway and increased the expression of p-CREB in the hippocampus of Aβ-induced AD mice, which might contribute to the neuroprotective effects of Ori.

In summary, our current study demonstrates that the administration of Ori attenuates the synaptic dysfunction and exerts significant neuroprotective effects in Aβ_1–42_-induced AD mice. In addition, the potential mechanism could be associated with the activation of the BDNF/TrkB/CREB signaling pathway. Therefore, Ori might be a potential drug that targets synapses for the treatment of AD.
